# Profil épidémiologique de l’insuffisance rénale chronique terminale dans la région de Sfax

**DOI:** 10.11604/pamj.2018.29.64.12159

**Published:** 2018-01-22

**Authors:** Yosr Chaabouni, Sourour Yaich, Azza Khedhiri, Mohamed Ali Zayen, Mahmoud Kharrat, Khawla Kammoun, Faical Jarraya, Mohamed Ben Hmida, Jamal Damak, Jamil Hachicha

**Affiliations:** 1 Service de Néphrologie, CHU Hedi Chaker, Sfax, Tunisie, Unité de Recherche de Pathologie Rénale, Faculté de Médecine, Sfax, Tunisie; 2 Service de Médecine Communautaire, CHU Hedi Chaker, Sfax, Tunisie; 3 Service de Néphrologie, CHU HediChaker, Sfax, Tunisie

**Keywords:** Épidémiologie, insuffisance rénale chronique terminale, néphropathie diabétique, Epidemiology, terminal chronic renal failure, diabetic nephropathy

## Abstract

**Introduction:**

L'insuffisance rénale chronique terminaleest un véritable problème mondial de Santé publique. En Tunisie, le coût de la prise en charge des patients dialysés pour l'année 2011 a dépassé les 90 millions de dinars (37000 Euro), soit près de 5% des dépenses globales de santé. Une meilleure connaissance du profil épidémiologique de l'insuffisance rénale chronique terminale va contribuer à l'élaboration et à l'évaluation des stratégies sanitaires visant à améliorer la prévention et la prise en charge de cette maladie. L'objectif de notre travail est de décrire le profil épidémiologique des cas incidents dans le gouvernorat de Sfax sur une période de 10 ans.

**Méthodes:**

Il s'agit d'une étude descriptive rétrospective allant de Janvier 2003 à Décembre 2012. Nous avons inclus les cas incidents d'insuffisance rénale chronique terminale dans le gouvernorat de Sfax.

**Résultats:**

Le diagnostic d'insuffisance rénale chronique terminale a été porté à 1708 cas. Il s'agit de 957 hommes et 751 femmes (sex-ratio = 1,27). L'âge moyen était de 58,4ans [10-100ans]. L'étude de l'évolution de l'âge moyen durant la période étudiée a montré une tendance vers la hausse avec un indice de corrélation positive (0,749) et p = 0,006. La principale néphropathie causale était la néphropathie diabétique (21,5%), avec une augmentation significative de sa fréquence d'une année à l'autre (un coefficient de corrélation positive (0,770) avec p = 0,009). L'hémodialyse était la technique de dialyse de choix, entreprise chez 96% des patients.

**Conclusion:**

Un registre national reste indispensable afin de mieux comprendre le profil épidémiologique de l'insuffisance rénale chronique terminale en Tunisie et de pouvoir améliorer sa prise en charge.

## Abstract (en)

**Introduction:**

Terminal chronic renal failure is a truly global public health problem. In 2011, the cost of management of patients on dialysis has surpassed 90 million dinars (37.000 euro) in Tunisia, nearly 5% of the overall health spending. A better knowledge of the epidemiological profile of terminal chronic renal failure contributes to the implementation and evaluation of health strategies aimed to improve prevention and disease management. This study aimed to describe the epidemiological profile of incident cases in the Sfax Governorate over the period of 10 years.

**Methods:**

We conducted a descriptive retrospective study over the period from January 2003 to December 2012. The incident cases of terminal chronic renal failure in the Sfax Governorate were included in the study.

**Results:**

The diagnosis of terminal chronic renal failure was made in 1708 cases: 957 men and 751 women (sex-ratio = 1.27). The average age was 58.4 years [10-100 years]. The study of the evolution of the average age during the study period showed a tendency to increase with positive correlation coefficient (0.749) and p = 0.006. The main causal nephropathy was diabetic nephropathy (21.5%), with a significant increase in its frequency from one year to the other (positive correlation coefficient (0.770) with p = 0.009). Hemodialysis was the dialysis technique of choice, performed in 96% of patients.

**Conclusion:**

A national registry is indispensable in order to better understand the epidemiological profile of terminal chronic renal failure in Tunisia and to improve its management.

## Introduction

L'insuffisance rénale chronique terminale (IRCT) est un véritable problème mondial de Santé publique, imposant une lourde prise en charge et un coût élevé. En Tunisie, le coût de la prise en charge des patients dialysés pour l'année 2011 a dépassé les 90 millions de dinars (37000 Euro), soit près de 5% des dépenses globales de santé. Cette situation tend à s'aggraver régulièrement vu la progression de la prévalence de l'IRCT qui a connu une ascension fulgurante ces dernières années [1]. Une meilleure connaissance du profil épidémiologique de l'IRCT va contribuer à l'élaboration et à l'évaluation de stratégies sanitaires visant à améliorer la prévention et la prise en charge de cette maladie. L'objectif de notre travail est de décrire le profil épidémiologique des cas incidents d'IRCT dans le gouvernorat de Sfax, Tunisie sur une période allant de janvier 2003 à Décembre 2012.

## Méthodes

**Type de l'étude:** Notre travail a été réalisé au service de néphrologie du CHU Hédi Chaker de Sfax, Tunisie. Il s'agit d'une étude descriptive rétrospective portant sur une série de 1708 cas incident sur une période de 10 ans allant de janvier 2003 à décembre 2012.

**Population à l'étude:** Nous avons inclus dans notre travail les cas incidents originaire du gouvernorat de Sfax ayant une IRCT.Le gouvernorat de Sfax est situé au Centre Est de la Tunisie et il comptait en 2012 une population de 955 500 habitants. Ce gouvernorat est divisé en 15 délégations. Sachant que tous les cas incidents d'IRCT passent obligatoirement par le service de néphrologie du CHU Hédi Chaker de Sfax. Tout patient hors le gouvernorat de Sfax a été exclu de notre étude.

**Recueil des données:** Nous avons utilisé pour la collecte des données les dossiers des malades suivis au service de néphrologie CHU Hédi Chaker de Sfax.

**Définitions opérationnelles des variables:** L'IRCT est définie par un débit de filtration glomérulaire strictement inférieur à 15 ml/min/1,73 m^2^ [2]; la néphropathie diabétique (ND) se définit par la présence persistante d'une macro albuminurie (excrétion urinaire d'albumine > 300 mg/24 heures) associée à une altération de la clairance à la créatinine en présence d'un diabète [3]; la néphropathie vasculaire (NVAS) regroupent des maladies hétérogènes caractérisées par une atteinte des vaisseaux rénaux. Théoriquement, le calibre des vaisseaux rénaux lésés détermine trois catégories de néphropathie vasculaire: la sténose athéromateuse des artères rénales, la néphroangiosclérose et la néphropathie vasculaire consécutive à l'artériosclérose [2]; les néphropathies glomérulaires chroniques (NGC) désignent des affections au cours desquelles les lésions histologiques touchent principalement les glomérules. Leur classification repose sur les données anatomopathologiques, incluant les lésions observées en microscopie optique et les résultats de l'examen en immunofluorescence. Les deux principaux signes glomérulaires sont la protéinurie et l'hématurie. Ces signes peuvent être associés à une hypertension artérielle et/ou à une insuffisance rénale [4]; les néphropathies interstitielles chroniques (NIC) regroupent une large variété de pathologies très diverses. Elles sont caractérisées par un tableau rénal qui traduit la dysfonction tubulaire et ont une évolution relativement lente. Indépendamment de la cause de déclenchement, les signes de maladies tubulointerstitielles sont l'atrophie tubulaire, la fibrose interstitielle et l'infiltration cellulaire [5]; les néphropathies héréditaires (NH) regroupent les néphropathies qui possèdent une prédisposition génétique.

**Etude statistique:** La saisie et l'analyse des données ont été réalisées en utilisant le logiciel SPSS dans sa 20^ème^ version.

**Etude descriptive:** Les variables quantitatives ont été décrites en utilisant les médianes, les diagrammes en boites à moustaches, les moyennes, l'écart type et les limites. Les variables qualitatives ont été décrites en utilisant les proportions.

**Etude analytique uni variée:** L'étude des associations entre les variables a été faite par les tests d'hypothèses. La comparaison des proportions a été réalisée par le test de « chi2 » de Pearson ou par le test exact de « Fisher ». Le test de Student a été utilisé pour la comparaison de deux moyennes de deux échantillons indépendants. Le test « Anova » a été utilisé pour la comparaison de plusieurs moyennes. Pour cela, le test de Levene a été utilisé pour tester l'homogénéité des variances. Dans le cas de l'hypothèse de variance inégale, le test de Welch a été utilisé et nous avons eu recours aux tests post hoc (Scheffé, LSD, Tuckey') pour voir les groupes qui différaient significativement. L'étude de tendance générale des séries chronologiques a été faite par le test des rangs de Spearman. Une série chronologique est constituée par une succession d'observations, sur un même sujet ou sur un même phénomène, régulièrement espacées dans le temps. Les séries chronologiques sont mensuelles, trimestrielles ou annuelles, parfois hebdomadaires, journalières, voire horaires (étude de trafic routier, trafic téléphonique) ou au contraire biennales, décennales (recensement de la population).Pour tous les tests réalisés, le seuil de signification a été fixé à 5%.

## Résultats

**Figure 1:**
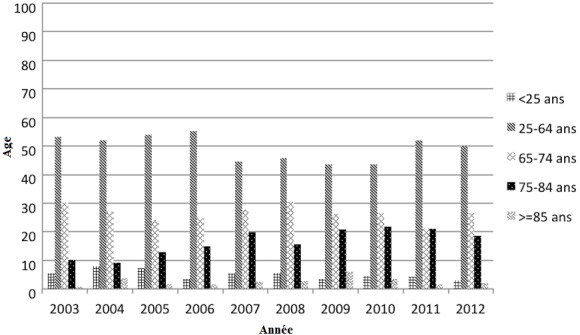
Répartition des patients par tranche d’âge en pourcentage et par année

**Figure 2:**
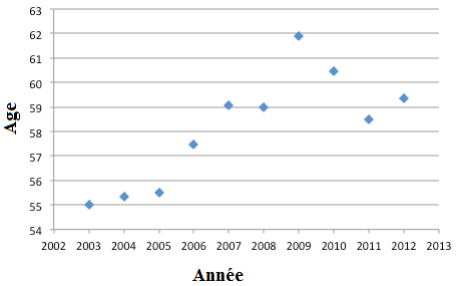
Evolution de l’âge moyen au moment du diagnostic de l’IRCT durant la période de l’étude (2003-2012)

1708 IRCT incidents ont été diagnostiqués durant la période de l'étude. L'incidence moyenne était de 170,8 cas/an avec des extrêmes allant de 109 à 190 cas/an. Cette incidence est en hausse, elle passe de 129,9 pmh en 2003 à 163,2 pmh en 2012 (Tableau 1). Nos patients sont répartis en 957 hommes et 751 femmes, soit un sex-ratio de 1,27. L'âge moyen de nos patients au moment du diagnostic de l'IRCT était de 58,4 ans avec des extrêmes allant de 10 à 100 ans. Dans 50% des cas, l'âge était entre 25 ans et 64 ans. Le pourcentage des patients dont l'âge entre 75 et 84 ans était en augmentation progressive, avec un pourcentage qui a passé de 10% en 2002 à 20% en 2012 (Figure 1). On remarque une différence significative des moyennes d'âge d'une année à une autre, test d'anova (p = 0,002). L'âge moyen est passé de 55 ans en 2002 à 59,4 ans en 2012. L'étude de l'évolution de l'âge moyen faite par le test de Spearman durant la période étudiée a montré une tendance vers la hausse avec un indice de corrélation positif (0,749) et p = 0,006 (Figure 2). La NDa été retrouvée dans 21,5% des cas. La NIC a été retrouvée dans 15% des cas. La NGCa été retrouvée dans 7,9% des cas. La NH et la NVAS étaient présentes respectivement dans 5,4% et 4,4% des cas. Enfin, la néphropathie initiale est restée indéterminée dans 45,8% des cas. La répartition des néphropathies initiales est restée la même pendant toute la période de l'étude ainsi que l'ordre de fréquence (Tableau 2). Pour la ND, on a trouvé un coefficient de corrélation positive (0,770) avec p = 0,009, soit une augmentation significative de sa fréquence. L'analyse de l'évolution de la fréquence des néphropathies indéterminées par le test de Spearman au cours de la période d'étude a montré un coefficient de corrélation négative (-0,709) avec p = 0,022 soit une diminution significative de sa fréquence. Cependant pour les autres types de néphropathie, il n'existait pas une variation statistiquement significative. L'hémodialyse est restée la technique la plus utilisée dans notre population. En effet, elle est observée chez 96% des patients durant toute la période. La dialyse péritonéale a été utilisée dans 2,6% des cas durant toute la période (Tableau 3). Le nombre de patients ayant bénéficié de la dialyse péritonéale était en augmentation progressive (Tableau 3). Ceci a été vérifié statistiquement par le test de Spearman et on a trouvé un coefficient de corrélation positive égal à 0,756 avec une p = 0,018.Dans notre série on a trouvé que le pourcentage de malade ayant bénéficié d'une greffe rénale préemptive était de 1,5% (25 cas) durant toute la période (Tableau 3).

**Tableau 1:** Evolution de l’incidence I’IRCT diagnostiqués dans le gouvernorat de Sfax, Tunisie durant la période de l’étude (2003-2012)

Année	Nombre d’habitants	Nombre de cas	Incidence par million d’habitants
2003	839 000	109	129,9
2004	849 700	163	191,8
2005	860 000	170	197,8
2006	881 000	190	215,6
2007	893 000	166	185,8
2008	905 000	186	205,5
2009	918 000	196	213,5
2010	931 000	187	200,8
2011	937 900	185	197,2
2012	955 500	156	163,2

**Tableau 2:** Evolution des néphropathies causales de l’IRCT dans le gouvernorat de Sfax, Tunisie durant la période de l’étude (2003-2012)

Néphropathie initiale	2003	2004	2005	2006	2007	2008	2009	2010	2011	2012	
NI (%)	49 (45%)	87 (53,4%)	84 (49,4%)	93 (48,9%)	71 (42,8%)	91 (48,9%)	94 (47,9%)	72 (38,5%)	75 (40,5%)	67 (42,9%)	
ND (%)	23 (21,1%)	21 (12,9%)	34 (20%)	33 (17,4%)	41 (24,7%)	38 (20,4%)	48 (24,5%)	42 (22,5%)	48 (25,9%)	40 (25,6%)	
NIC (%)	18 (16,5%)	28 (17,2%)	21 (12,4%)	26 (13,7%)	25 (15,1%)	25 (13,4%)	27 (13,8%)	30 (16%)	32 (17,3%)	23 (14,7%)	
NG (%)	12 (11%)	14 (8,6%)	20 (11,8%)	12 (6,3%)	12 (7,2%)	14 (7,5%)	8 (4,1%)	15 (8%)	16 (8,6%)	14 (9%)	
NF (%)	1 (0,9%)	10 (6,1%)	8 (4,7%)	16 (8,4%)	12 (7,2%)	9 (4,8%)	9 (4,6%)	11 (5,9%)	10 (5,4%)	6 (3,8%)	
NVAS (%)	6 (5,5%)	3 (1,8%)	3 (1,8%)	10 (5,3%)	5 (3%)	9 (4,8%)	10 (5,1%)	17 (9,1%)	4 (2,2%)	6 (3,8%)	
Total	109 (100%)	163 (100%)	170 (100%)	190 (100%)	166 (100%)	186 (100%)	196 (100%)	187 (100%)	185 (100%)	156 (100%)	

NI, Néphropathie indéterminée ; ND, Néphropathie diabétique ; NIC, néphropathie interstitielle chronique ; NG, Néphropathie glomérulaire ; NF, Néphropathie familiale ; NVAS, Néphropathie vasculaire

**Tableau 3:** Evolution de la prise en charge de l’IRCT à l’hôpital Hédi Chaker de Sfax, Tunisie durant la période de l’étude (2003-2012)

Méthode	2003	2004	2005	2006	2007	2008	2009	2010	2011	2012
HD (%)	109 (100%)	158 (96,9%)	166 (97,6%)	181 (95,2%)	163 (98,2%)	174 (93,5%)	183 (93,3%)	179 (95,7%)	178 (96,2%)	148 (94,8%)
DP (%)	0 (0%)	3 (1,8%)	3 (1,7%)	5 (2,6%)	1 (0,6%)	5 (2,7%)	10 (5,1%)	6 (3,2%)	5 (2,7%)	6 (3,8%)
GR préemptive (%)	0 (0%)	2 (1,2%)	1 (0,6%)	4 (2,1%)	2 (1,2%)	7 (3,8%)	3 (1,5%)	2 (1%)	2 (1,1%)	2 (1,3%)

DP: Dialyse péritonéale, GR préemptive: Greffe rénale préemptive, HD: Hémodialyse

## Discussion

L'insuffisance rénale chronique représente un problème majeur de santé publique à l'échelle mondiale, du fait de la mortalité élevée, du coût important des traitements requis ainsi que de la baisse considérable de la qualité de vie. L'IRCT constitue de ce fait une charge socio-économique lourde. En effet, le nombre de nouveaux patients qui arrivent au stade terminal est en augmentation progressive. Dans notre pays, peu de données existent concernant l'épidémiologie de l'IRCT vu l'absence d'un registre national depuis l'étude multicentrique de Ben Maiz et al en 2008, ayant montré une ascension fulgurante de l'incidence de l'IRCT en Tunisie qui est passée de 13 par million d'habitants (pmh) en 1986 à 133 pmh en 2008 [1]. En dépit du caractère monocentrique de notre étude, elle apporte de récentes données épidémiologiques de cette pathologie lourde et onéreuse. Elle a montré que l'incidence de l'IRCT est également en hausse dans le gouvernorat de Sfax (de 129,9 pmh en 2003 à 163,2 pmh en 2012). Selon le registre de l'EDTA de 2013, l'incidence globale de l'IRCT dans 34 différents pays est de 112 pmh [6], alors qu'elle était 109,9 pmh en 1997 [7]. Cette incidence varie considérablement entre les différents pays. Les données de 2013 montrent qu'elle est beaucoup plus élevée en Grèce (216 pmh) par rapport à la Russie (50 pmh) et l'Ukranie (30 pmh) [6]. Cette incidence est également en hausse au fil des années pour la majorité des pays. Les variations de l'incidence d'un pays à l'autre peuvent être expliquées par la différence dans le profil étiologique de l'IRCT, la différence de survie des patients atteints de maladies cardio-vasculaires et de diabète, l'accès au traitement de suppléance qui est en effet plus difficile dans les régions rurales ou en grande précarité. Cette variation peut être aussi due à une abstention thérapeutique de la part des médecins généralistes ou des néphrologues chez des patients dont le bénéfice de la dialyse est incertain. Enfin, elle peut être aussi due à la qualité des registres et la différence de prise en charge des patients atteints d'IRCT [8]. Dans notre étude, l'âge moyen des patients au moment du diagnostic de l'IRCT était de 58,4 ans, allant de 55 ans en 2002 à 59,4 ans en 2012 avec une augmentation statistiquement significative d'une année à une autre (p=0,002 et un indice de corrélation positive=0,749). Une nette augmentation de la proportion des patients âgés de plus de 75 ans a été notée, avec un pourcentage qui est passé de 10% en 2002 à 20% en 2012. Nos données sont similaires à celles du registre de l'EDTA, où l'âge moyen global était de 58,2 ans en 2012 [9].

En étudiant le profil étiologique des patients en IRCT, la ND paraît comme l'étiologie la plus fréquente dans notre série. Toutefois, le pourcentage des néphropathies d'origine indéterminée reste très élevé dans notre série. Ceci peut être dû à des imprécisions nosologiques dans la description des maladies rénales ainsi que le retard de consultation chez le néphrologue, le diagnostic étiologique étant difficile à un stade avancé de l'IRC. Cependant, l'allure décroissante de la fréquence des néphropathies indéterminée, avec un coefficient de corrélation négative (-0,709) et p = 0,022, est un bon signe de l'amélioration de la prise en charge de IRC à un stade précoce, essentiellement en faveur de la ND, qui est la seule néphropathie dans notre étude ayant eu une augmentation significative de sa fréquence (coefficient de corrélation positive (0,770) avec p = 0,009). Ceci est aussi valable pour le Japon, comme pour les autres pays asiatiques, où le diabète constitue la première cause d'IRCT [10]. Aux états Unis, la ND est retrouvée comme cause d'IRCT dans 44% des cas en 2008 [11]. Elle est 5 fois plus élevée que celle observée en Europe selon le registre de l'EDTA 2013. Toutefois, malgré l'augmentation linéaire du nombre de diabétiques aux états unis et en Europe [12, 13], l'incidence de l'IRCT secondaire à la ND est restée stable dans ces pays durant la dernière décennie [11]. Ceci témoigne probablement d'une meilleure prise en charge de la ND au cours des dernières décennies du fait de la généralisation du contrôle glycémique strict et de l'utilisation des bloqueurs du système rénine angiotensine et le bon équilibre tensionnel [12].

Par ailleurs, la NVAS a représenté la fréquence la plus basse dans notre série en comparaison avec les autres néphropathies initiales. Cette fréquence est restée stable au cours du temps, contrairement aux pays développés, où la NVAS constitue une cause croissante de mise en dialyse [14]. Les données européennes vont dans le même sens puisque les NVAS représentaient 18,1% des patients bénéficiant d'un traitement de suppléance en 2013 contre 12,6 % en 1995 [6, 15]. Dans notre étude, le traitement de suppléance le plus utilisé était l'hémodialyse, entreprise chez 96% des patients durant toute la période. La dialyse péritonéale reste une technique très peu utilisée dans notre centre. Toutefois, l'étude de son évolution montre qu'elle est en train d'augmenter d'une manière significative au cours du temps. Il va de même pour les pays européens, où l'hémodialyse reste le traitement de suppléance de choix, avec une incidence de 98% de l'ensemble des patients en dialyse en 2013 selon le registre de l'EDTA, contre 17,5% seulement en dialyse péritonéale. Ces pourcentages sont variables d'un pays à l'autre, selon le système de santé et la politique de soins de chaque pays. Le recours à la DP en 2012 varie entre 3% des patients en traitement de suppléance en Bosnie à 32% au Suède [9]. Cette technique reste limitée, mis à part ses contre-indications, par les modes de financement du traitement, le type du système de santé et la qualité des structures sanitaires. Dans notre étude, le nombre de malade ayant bénéficié d'une greffe préemptive est très réduit et ne présente que 1,5%. Le manque de greffons disponibles en constitue la limite majeure et persiste malgré la mise en œuvre dans notre pays d'un centre national pour la promotion et la transplantation d'organes et l'instauration d'une loi claire facilitant le prélèvement d'organes à partir de morts encéphaliques [16]. Bien que le bénéfice d'une greffe préemptive ait été clairement établi, essentiellement dans l'amélioration de la qualité de vie et la réduction du cout de prise en charge des IRCT [17], cette modalité thérapeutique reste encore très limitée dans plusieurs pays développés. Selon le registre de l'EDTA de 2013, le pourcentage de la greffe préemptive varie de 0% en Grèce et en Bosnie, à 15% et 16% respectivement au Norvège et aux Pays-Bas [6]. Ce faible développement de la greffe préemptive est expliqué essentiellement par la pénurie de greffons et par le retard observé dans l'inscription des patients insuffisants rénaux sur la liste d'attente.

## Conclusion

Notre étude a montré une élévation considérable et régulière de l'incidence de l'IRCT dans le gouvernorat de Sfax avec une nette augmentation de la proportion de personnes âgées vu l'amélioration de la prise en charge et la qualité des soins ainsi que l'allongement de l'espérance de vie. Une prédominance de la ND a été notée, bien que le pourcentage de néphropathies d'origine indéterminée reste élevé. Devant le nombre réduit de greffes préemptives, la prise en charge des patients en IRCT passe essentiellement par l'hémodialyse. En Tunisie, l'IRCT représentent un vrai problème de santé publique nécessitant une prise charge globale faisant intervenir l'ensemble des acteurs de la sante publique.

### Etat des connaissances actuelles sur le sujet

- L'IRCT est un véritable problème mondial de Santé publique. En Tunisie, le coût de la prise en charge des patients dialysés pour l'année 2011 a dépassé les 90 millions de dinars (37000 Euro), soit près de 5% des dépenses globales de santé;
- Dans notre pays, peu de données existent concernant l'épidémiologie de l'IRCT vu l'absence d'un registre national.

### Contribution de notre étude à la connaissance

- Une meilleure connaissance du profil épidémiologique de l'IRCT va contribuer à l'élaboration et à l'évaluation des stratégies sanitaires visant à améliorer la prévention et la prise en charge de cette maladie;
- La principale néphropathie causale de l'IRCT était la ND (21,5%), avec une augmentation significative de sa fréquence d'une année à l'autre (un coefficient de corrélation positive (0,770) avec p=0,009);
- Une meilleure prise en charge de la ND dans notre région est primordiale parle contrôle glycémique strict, l'utilisation des bloqueurs du système rénine angiotensine et le bon équilibre tensionnel pour prévenir la progression de la ND à un stade terminal.

## Conflits d’intérêts

Les auteurs ne declarent aucun conflit d’'intérêts.
